# Recent Advances in Coronary Artery Bypass Grafting Techniques and Outcomes: A Narrative Review

**DOI:** 10.7759/cureus.45511

**Published:** 2023-09-18

**Authors:** Vaishnavi s Thakare, Nikhil G Sontakke, Praful Wasnik, Deepika Kanyal

**Affiliations:** 1 Hospital Administration, Jawaharlal Nehru Medical College, Datta Meghe Institute of Higher Education and Research, Wardha, IND; 2 Health Sciences, Jawaharlal Nehru Medical College, Datta Meghe Institute of Higher Education and Research, Wardha, IND; 3 Medicine, Jawaharlal Nehru Medical College, Datta Meghe Institute of Higher Education and Research, Wardha, IND

**Keywords:** heart, treatment modality, surgery, percutaneous coronary intervention, coronary revascularization, coronary bypass

## Abstract

Coronary artery bypass grafting (CABG) has witnessed remarkable progress in recent years, driven by innovative techniques and refined approaches that have transformed patient outcomes and treatment paradigms. This review article comprehensively explores the latest advances in CABG techniques and their consequential impacts on patient outcomes. The advent of minimally invasive CABG techniques has revolutionized traditional surgical approaches. Robotic-assisted surgery and small thoracotomy methods offer reduced invasiveness, yielding shorter recovery times and improved patient satisfaction. Integrating CABG with percutaneous coronary intervention (PCI), hybrid procedures have emerged as a versatile strategy, providing tailored treatment solutions for complex coronary anatomies. The paradigm shift to off-pump CABG, which preserves the beating heart during surgery, has shown promise in reducing perioperative complications and neurocognitive deficits. Advances in graft selection, particularly the utilization of arterial grafts such as the internal thoracic artery and radial artery, have significantly enhanced graft patency rates and long-term survival. Adjunctive technologies, such as intraoperative imaging and graft flow assessment, have bolstered the precision of CABG procedures. Pharmacological agents have demonstrated their potential to improve graft outcomes. Surgical decision-making is now optimized based on patient characteristics and disease severity owing to the development of patient selection and risk stratification tools. Long-term results have also significantly improved. Patients undergoing CABG have higher survival rates, less angina, and better quality of life. Developing more resilient grafts through tissue engineering, using bioresorbable materials in graft fabrication, and using gene therapy to improve graft patency and overall cardiac recovery are all exciting future research directions. This review's summary of current developments in CABG procedures highlights their profoundly positive effects on patient outcomes. These developments can change the face of cardiovascular care by giving clinicians more tools to treat coronary artery disease (CAD) and enhance patients' quality of life.

## Introduction and background

In poorer regions of the planet, this disease does not represent a primary cause. Even in well-developed countries, cerebrovascular disease (as in the form of stroke ) also represents an equal cause of morbidity and mortality [1]. The care of coronary artery disease (CAD) still relies heavily on coronary artery bypass grafting (CABG), which offers a tried-and-true method for restoring myocardial blood flow and enhancing patient outcomes. Toward the turn of the 20th century, mortality (in age-adjusted participants) decreased as a result of scientific advances in our understanding of the pathophysiology of CAD [1,2]. Cardiovascular surgery has changed dramatically over the past few decades due to a wide range of CABG procedures and postoperative care innovations that have improved patient outcomes, decreased morbidity, and raised quality of life. CABG has seen substantial development since its beginnings in the 1960s when cardiopulmonary bypass and sternotomy were combined. Recent efforts aim to improve patient-centered outcomes, increase graft patency, and lessen surgical trauma [3,4]. People over the age of 35 die from CAD in developing and industrialized nations, with the percentage in Western countries approaching 50% [5,6]. The World Health Organisation predicts that, by 2024, the global burden will amount to 1.5 billion disabled persons (years lost due to disability, bad health, or death) [7]. According to estimates, 42.5 million people in the United States America (USA) alone will suffer from or pass away from CAD and its consequences in 2023 [8]. One of the most common and expensive operations in the USA continues to be coronary revascularization, either through percutaneous coronary intervention (PCI) or CABG, with Medicare inpatient payments reaching $6.7 billion yearly [9,10]. A left internal mammary artery (LIMA) graft to the left anterior descending coronary artery (LAD) is a common component of CABG in the USA. Numerous studies have highlighted the advantages of surgically omitting the LAD in favor of the LIMA graft. The LIMA graft not only outperforms all other treatments for CAD in terms of improving life expectancy and symptom reduction, but excellent data from Tatoulis et al. presented that these advantages last the entire lifespan of the graft and that graft patency beyond 10 and 15 years is not uncommon [4,11]. The influence of current developments in CABG procedures on patient outcomes is examined in this review paper.

## Review

Search methodology

**Figure 1:**
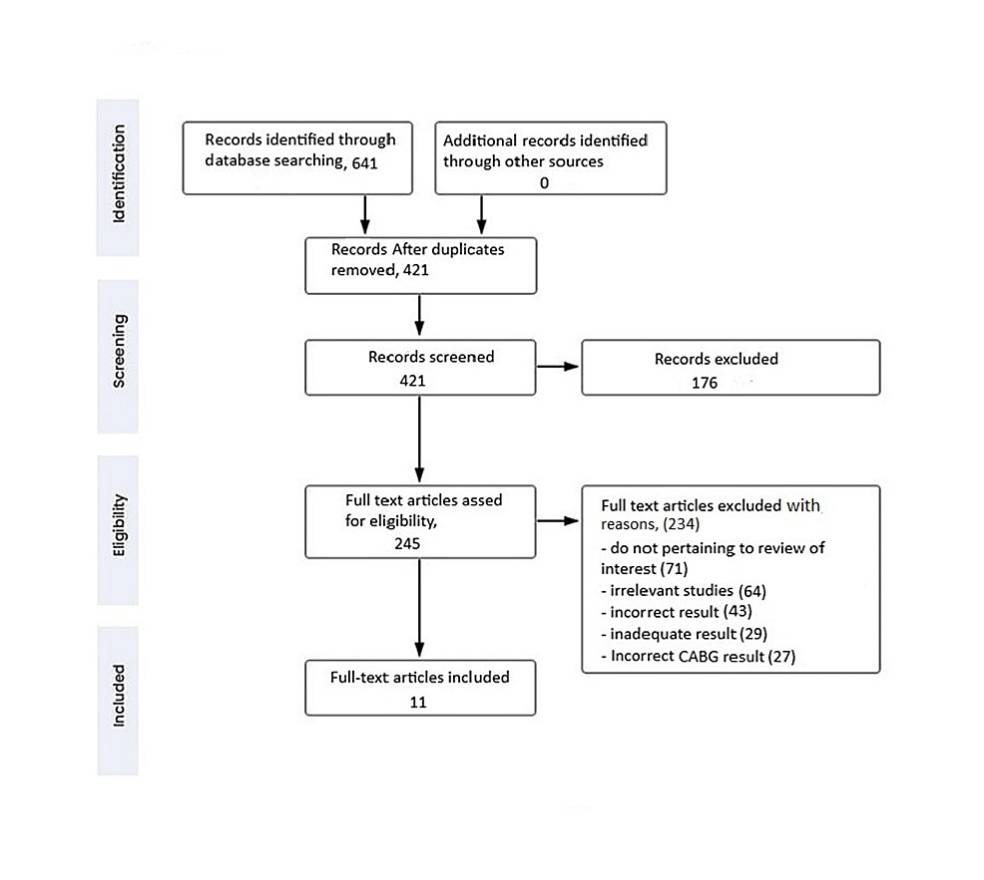
Prisma flow chart of search strategy Own creation. Source [celegence]

We undertook a search through PubMed Central in March 2023 using keywords such as "coronary bypass," "percutaneous coronary intervention," "coronary revascularization," "heart," and "surgery"((( coronary bypass [Title/Abstract]) OR ("coronary bypass" [MeSH Terms]), ("percutaneous coronary intervention" [Title/Abstract])) OR ("percutaneous coronary intervention" [MeSH Terms]), (("coronary revascularization" [Title/Abstract]) OR ("coronary revascularization" [MeSH Terms]) AND ("heart" [Title/Abstract]) OR ("heart" [MeSH Terms]) (Table 1). One reviewer independently checked the papers retrieved based on title and abstract against the inclusion criteria before moving on to the full texts. Another reviewer also reviewed 20% of these studies to validate inclusion studies. The selection of studies (Figure 1) depended on the following inclusion criteria: (1) coronary artery bypass grafting, (2) patient with heart problems, (3) heart surgery, (4) English language, and (5) systematic reviews. The following were the exclusion criteria: (1) case study, (2) non-English language articles, (3) opinion articles, (4) technical reports, and (5) surveys. Figure 1 shows the PRISMA flow diagram for articles search.

**Table 1:** Inclusion and exclusion criteria for the searched articles

Parameters	Inclusion Criteria	Exclusion Criteria	Keywords
Location	International	None	N/A
Language	English	Non-English languages	English language only
Time	Any	None	N/A
Population	Studies which include CABG procedure	Studies that focus on other surgeries	((coronary bypass [Title/Abstract]) OR ("coronary bypass" [MeSH Terms]
Phenomena/ target	Study concern about recent advances in coronary artery bypass grafting techniques and outcomes	Not concerned with the other diseases and surgery related to cardiovascular diseases	("percutaneous coronary intervention" [Title/Abstract])) OR ("percutaneous coronary intervention" [MeSH Terms]), (("coronary revascularization" [Title/Abstract]) OR ("coronary revascularization" [MeSH Terms]) AND ("heart" [Title/Abstract]) OR ("heart" [MeSH Terms])
Study	Full-text articles, patients with heart problems, systematic review, English language	Studies that do not include full-text articles, patients with heart problems, systematic reviews, English language	N/A

Minimally invasive CABG techniques

One of the most critical changes to CABG has been the introduction of minimally invasive techniques. As potential alternatives to traditional CABG, minimally invasive direct coronary artery bypass (MIDCAB) and robotic-assisted CABG have evolved. These methods use tiny incisions, minimizing surgical trauma and hastening the healing process after surgery. Small thoracotomies are used in MIDCAB to access the desired coronary arteries. On the other hand, robotic-assisted CABG uses robotic arms to make accurate anastomoses on a beating heart. These procedures, developed by organizations such as the Cleveland Clinic and Mayo Clinic, have demonstrated promise in raising patient satisfaction and comfort levels while retaining reasonable graft patency rates [12,13]. Treatment for CAD is revolutionized by minimally invasive CABG techniques. A sternotomy and cardiopulmonary bypass are required during traditional CABG surgery, which results in lengthy hospital stays and a drawn-out recovery. Contrarily, minimally invasive CABG procedures are designed to lessen the stress of surgery, improve patient outcomes, and hasten recovery. In a randomized trial contrasting MIDCAB with traditional CABG, Kappert et al. found that MIDCAB resulted in lower morbidity and quicker recovery [14].

Hybrid approaches: integrating techniques for optimal outcomes

Hybrid techniques that combine CABG with PCI result from the interaction between cardiac surgery and interventional cardiology. Combining the advantages of both procedures, this strategy aims to treat left primary coronary lesions and complex multivessel disease. With hybrid revascularization, a customized approach is possible in which the patient's anatomical and clinical parameters determine the best course of action. Clinical studies, such as the "EXCEL" trial, have shown that hybrid treatments are non-inferior to traditional CABG, giving patients a customized revascularization plan [15]. In CABG, hybrid procedures advantageously combine surgical and interventional cardiology methods. The procedure's invasiveness is reduced owing to its integration, among other benefits. By combining traditional surgical techniques with minimally invasive procedures, patients benefit from fewer incisions, less blood loss, and quicker recovery times. Hybrid procedures also allow one to deal with complex cases that might not be suitable for standard techniques alone [16]. This method encourages individualized patient care by adjusting therapies to each patient's anatomy and pathology. Combining CABG with PCI is a popular hybrid procedure [15]. When patients have multivessel disease, surgeons can use PCI for less severe lesions while doing CABG on the most important coronary artery. This prevents the necessity for a full sternotomy while maintaining both techniques' advantages. According to research papers, hybrid strategies frequently lead to increased cardiac perfusion, decreased morbidity, and shorter hospital stays, demonstrating their potential to enhance patient outcomes [17]. Although mixed techniques have promising advantages, there are still some difficulties. One significant consideration is the need for effective collaboration between cardiovascular surgeons and interventional cardiologists. Seamless communication and shared decision-making are essential to determine the most suitable approach for each patient. Moreover, the technical complexity of hybrid procedures demands a high level of expertise and training from the medical team. Complications can arise if the team lacks experience in both surgical and interventional aspects [18]. The realm of hybrid approaches in CABG continues to evolve with ongoing advancements in medical technology. Integrating robotic-assisted surgery, three-dimensional imaging, and more refined catheter-based interventions can further refine outcomes and reduce procedural risks. Additionally, as patient populations become increasingly diverse, tailoring hybrid strategies to accommodate various demographics and risk profiles will be essential [15,19-21].

Off-pump CABG: preserving cardiac function

A significant turning point in cardiac surgery was the introduction of off-pump CABG, in which the heart continues to beat while the graft is implanted. The technique was created to reduce the likelihood of adverse effects from cardiopulmonary bypass, including systemic inflammation and neuropsychological deficits. Off-pump CABG aims to reduce issues, improve patient outcomes, and speed up postoperative recovery. Studies assessing the effectiveness and safety of off-pump CABG have focused on high-risk patients [22,23]. To improve patient outcomes and quality of life, cutting-edge procedures have revolutionized the area of cardiac surgery. Off-pump CABG, a surgery intended to preserve heart function while treating coronary artery disease, is a fantastic achievement. Off-pump CABG is a novel strategy that has the potential to revolutionize cardiac surgery because it promises to reduce problems and enhance recovery [24]. It differs from the traditional on-pump technique in that it performs surgery without momentarily stopping the heart and connecting it to a heart-lung machine. Additionally, it allows the surgeon to work on a beating heart while avoiding restricted or obstructed coronary arteries so that can continue to pump blood. The potential to maintain heart function is the most notable benefit of this crucial differentiation [22,25]. In cardiac surgery, preserving heart function is of utmost importance. Off-pump CABG minimizes the risk of myocardial injury and lessens the possibility of postoperative problems by avoiding using the heart-lung machine, which briefly pauses blood flow and stresses the heart. According to research, patients who undergo off-pump CABG frequently experience fewer cases of atrial fibrillation, stroke, and neurocognitive deficits than those who receive the standard on-pump surgery [26]. The advantages of off-pump CABG also include quicker recovery times and reduced hospital stays. Patients typically suffer less pain and resume daily activities more quickly with less invasive procedures and cardiac trauma. This is a significant benefit for people who want to return to their usual lives as soon as possible. The technique's usefulness for high-risk patients further emphasizes its importance [27]. Off-pump CABG is a feasible alternative for individuals who would be viewed as poor candidates for the standard operation due to comorbidities or impaired heart function. For patients more vulnerable to surgical problems, off-[ump CABG offers a safer surgical option by reducing the stress on the heart [28]. Howeer, off-pump CABG does have specific difficulties. A high level of surgical knowledge and proficiency is required to perform the procedure on the active, beating heart. Accuracy is essential to guarantee successful grafting and prevent problems such as graft failure or insufficient revascularization [29]. With its emphasis on maintaining heart function and enhancing patient outcomes, CABG offers a transformational approach to cardiac surgery. This method minimizes myocardial injury, lowers postoperative problems, and speeds up recovery times by doing surgery on a beating heart, instead of using a heart-lung machine [23]. It is a promising development since it can be used with high-risk patients and potentially alter conventional paradigms in cardiac surgery. However, to overcome the difficulties of operating in a dynamic cardiac environment, its execution requires a high level of surgical experience [30]. As the medical field continues to evolve, off-pump CABG stands as a testament to the potential for innovation to reshape traditional practices and enhance the lives of individuals affected by coronary artery disease. Table 2 discusses the overview of techniques use in CABG and their outcomes. 

**Table 2:** Short summary of techniques used in CABG

Sr.no	Technique	Description	Outcomes
1)	Minimally Invasive CABG	Involves smaller incisions, often using robotics and specialized instruments for bypass grafting.	Reduced pain, shorter hospital stay, quicker recovery.
2)	Off-Pump CABG	Bypass grafts performed while heart still beating, avoiding use of heart-lung machine.	Lower risk of stroke and cognitive issues.
3)	Total Arterial CABG	Use of arterial grafts (e.g., internal mammary arteries) instead of veins, enhancing graft longevity and patient outcomes.	Improved long term graft patency, reduced cardiac events.
4)	Hybrid CABG Procedures	Combination of CABG with other interventions, such as percutaneous coronary intervention (PCI).	Tailor approach to address complex cases, improved symptomatic relief.
5)	Robotic-Assisted CABG	Surgeon controls robotic arms with enhanced dexterity to perform bypass grafting.	High precision, reduced trauma, shorter recovery time.
6)	Gene Therapy for Grafts	Genetic modification of grafts to enhance their patency and resistance to atherosclerosis.	Improved graft integration, reduced re-stenosis rate.
7)	Advanced Imaging Techniques	Integration of advanced imaging like intraoperative angiography and 3D mapping for precise grafting.	Enhanced surgical planning, accurate graft placement.
8)	Personalized Medicine	Tailoring CABG techniques based on patient's unique characteristics and genetic makeup.	Improved patient outcomes, reduced complications.

Advancements in graft selection

The choice of graft in CABG surgery still significantly impacts long-term results. Due to their higher patency rates compared to venous grafts, arterial grafts, particularly those from the internal thoracic artery (ITA) and radial artery, have become more popular. The ITA, an endothelialized conduit, demonstrates excellent long-term patency due to its resistance to atherosclerosis. The use of radial artery grafts, once considered challenging, has become more prevalent due to refinements in surgical technique and patient selection criteria [31-33]. Advancements in graft selection in CABG have revolutionized the field of cardiac surgery, enhancing patient outcomes and redefining surgical strategies. This review highlights the remarkable progress in graft selection techniques, underscoring their pivotal role in improving long-term graft patency and overall cardiac function [34]. The internal mammary artery and saphenous vein have historically served as the main grafting conduits. However, recent studies have demonstrated the advantages of arterial grafts, notably using the radial and left internal mammary arteries. The LIMA has emerged as the gold standard for grafting the left anterior descending artery due to its high long-term patency rates and physiological resemblance to the coronary artery. Previously an underutilized alternative, anastomosing the radial artery to routes other than the left anterior descending vessels produces outstanding results [35]. In addition, the development of hybrid methods mixing arterial and venous grafts, such as LIMA-to-LAD and radial artery-to-non-LAD grafting, is a result of technological improvements. These methods increase graft patency while reducing surgical intrusion. Additionally, real-time evaluation of graft performance is possible due to intraoperative imaging tools, including intraoperative angiography and Doppler flowmetry, which help surgeons make better graft selection selections and take quick corrective action [36]. Genomic and proteomic research have also paved the way for personalized graft selection, identifying patient-specific factors influencing graft success. By tailoring graft choices based on these factors, surgeons can optimize outcomes and reduce the likelihood of reoperation [37]. The evolution of graft selection in CABG reflects a paradigm shift towards arterial dominance and personalized medicine. Incorporating arterial grafts, hybrid procedures, and cutting-edge imaging technologies has significantly elevated the success rates of CABG surgeries. While challenges such as graft vasospasm and the availability of suitable conduits persist, ongoing research holds promise for even more refined graft selection techniques. As cardiac surgery embraces these advancements, patients can anticipate improved quality of life and extended longevity post-CABG [38].

Adjunctive technologies and patient selection

Integrating adjunctive technologies, such as intraoperative imaging and graft flow assessment, has contributed to refining CABG procedures. Intraoperative imaging techniques, including intraoperative angiography and indocyanine green fluorescence imaging, allow for real-time evaluation of graft patency. Graft flow assessment, often employing transit-time flowmetry, enhances intraoperative decision-making and graft quality assessment [39]. Moreover, patient selection algorithms have evolved to consider factors beyond anatomical suitability, encompassing individual patient characteristics and comorbidities.

Long-term outcomes and quality of life

Beyond perioperative outcomes, CABG's impact on long-term survival and quality of life is paramount. Patients undergoing CABG experience improved survival rates compared to medical therapy alone. Additionally, CABG significantly relieves angina, enhancing quality of life and functional capacity [40]. Studies consistently demonstrate that CABG substantially improves long-term survival rates and reduces cardiovascular events. The procedure effectively restores blood flow to the ischemic myocardium, alleviating symptoms and enhancing overall heart function. Long-term follow-ups reveal decreased mortality and lower rates of angina, bolstering the case for CABG's effectiveness as a durable treatment option [41]. Quality of life assessments post-CABG unveil a multifaceted picture. Patients commonly experience relief from chest pain, reduced limitations in physical activities, and improved psychological well-being due to enhanced cardiac function. However, factors such as preoperative health status, comorbidities, and postoperative complications can influence the degree of quality-of-life improvement [40]. These long-term benefits underscore CABG's role in effectively addressing the chronic nature of CAD.

Table 3 discusses the characteristics of article included in review. 

**Table 3:** Characteristics of articles included in the review

Author	Year	Country	Findings
Doğan et al. [12]	2005	Germany	Robotic surgery, involving beating and arrested hearts for endoscopic revascularization, is still in development, despite high costs and limited patient access.
Giambruno et al. [13]	2018	USA	In some individuals, robot-assisted coronary artery bypass grafting provides a safe, practical, and successful alternative to conventional coronary artery bypass grafting.
Caracciolo et al. [16]	1995	USA	The report shows that CABG surgery prolongs life in most clinical and angiographic subgroups in patients with diseases, but not in those with normal systolic function or significant right coronary artery stenosis.
Leacche et al. [17]	2013	USA	Hybrid coronary revascularization is a safe alternative to bypass grafting in multivessel coronary artery disease, but is superior in high-risk patients with complex coronary artery disease.
Rab et al. [19]	2012	USA	Hybrid coronary revascularization (HCR) for left main (LM) coronary disease is a feasible alternative to CABG and unprotected LM percutaneous coronary intervention (PCI). This approach combines the long-term durability of a LIMA-LAD bypass with the less invasive option of PCI in non-LAD targets with DES
Halkos ME et al, [21]	2011	USA	Hybrid coronary revascularization (HCR) offers a minimally invasive treatment for multivessel coronary artery disease, with comparable in-hospital and midterm outcomes to traditional off-pump coronary artery bypass grafting (OPCAB), warranting further investigation.
Sharoni et al. [22]	2006	Israel	For patients with impaired left ventricular function who might benefit from the off pump approach, OPCAB is a practicable and potentially helpful therapy.
Cleveland Jr et al. [25]	2001	USA	Off-pump coronary artery bypass grafting (CABG) is associated with lower mortality and morbidity after surgery and, in some patients, may be more efficient than traditional CABG.
Arom et al. [27]	2000	USA	The OPCAB approach is suitable for multivessel coronary artery bypass in patients with depressed left ventricular function, with success attributed to careful intraoperative detail and hemodynamic management.
Cartier et al. [30]	2000	Canada	Off-pump complete coronary artery revascularization is a viable alternative to conventional operations in most patients, resulting in good results due to experience, rigorous technique, and adequate stabilization.
Rodés-Cabau et al. [32]	2009	Canada	When compared to medical treatment alone, paclitaxel-eluting stents stenting of mild lesions in elderly saphenous vein graft slows the course of the disease and significant adverse cardiac events at 1-year follow-up.

## Conclusions

The evolution of CABG techniques from traditional approaches to minimally invasive procedures, hybrid interventions, off-pump strategies, and refined graft selection has redefined the landscape of CAD management. These advances have improved patient outcomes, reduced morbidity, and a broader arsenal of tools for cardiovascular surgeons. The subsequent sections of this review delve into the intricacies of each advancement, providing an in-depth analysis of the evidence, clinical implications, and future directions in the field of CABG. By fostering a comprehensive understanding of these advancements, this review aims to equip clinicians and researchers with the knowledge required to make informed decisions in the evolving realm of CABG.
